# Portable Low-Cost Sensors for Environmental Monitoring in China: A Comprehensive Review of Application, Challenges, and Opportunities

**DOI:** 10.3390/s26010085

**Published:** 2025-12-22

**Authors:** Chunhui Yang, Ruiyuan Wu, Yang Zhao, Jianbang Xiang

**Affiliations:** School of Public Health (Shenzhen), Sun Yat-sen University, Shenzhen 518107, China; yangchh28@mail2.sysu.edu.cn (C.Y.); wury28@mail2.sysu.edu.cn (R.W.)

**Keywords:** low-cost, sensor, monitor, environmental monitoring, air pollution

## Abstract

**Highlights:**

**What are the main findings?**

- Field applications of low-cost sensors in China are heavily skewed towards air and noise pollution, leaving critical areas like soil and biological contamination largely unexplored.
- The current research is predominantly limited by small-scale studies, short durations, and insufficient validation of sensor reliability.

**What is the implication of the main finding?**

- The identified gaps underscore an urgent need to extend the application of low-cost sensors to under-investigated environmental domains, which is essential for comprehensive multi-pollutant exposure assessment.
- This review provides a critical framework for future research, highlighting that overcoming challenges in sensor accuracy, portability, and data integrity is fundamental to achieving large-scale, reliable monitoring.

**Abstract:**

Accurate environmental monitoring in outdoor and indoor settings is critical for exposure assessment in environmental and public health research. Conventional methods, predominantly relying on high-end instruments or laboratory analyses, face limitations in real-world applications due to their high cost and inflexibility. Recent advances in low-cost sensor technologies have enabled more adaptable monitoring. This study systematically reviews research utilizing low-cost sensors for environmental monitoring in real-world settings across China. A literature search was performed using the Web of Science database, resulting in the inclusion of 43 eligible studies out of 31,003 initially identified records. These studies primarily investigated air pollution (17 studies), noise (14), light (7), and water pollution (5). Results reveal that air and noise pollution were the most extensively examined factors. Nevertheless, the reviewed studies exhibited notable shortcomings, including limited geographical/thematic coverage, inadequate reliability validation, small sample sizes (typically under 100 participants), and short durations (often under one month). This review discusses these challenges and suggests future research directions. By synthesizing current practices and identifying gaps, this work offers valuable insights to guide the design of future sensor-based environmental monitoring projects and inform the selection of suitable sensors.

## 1. Introduction

Despite general improvements in environmental quality over the past few years, excessive environmental exposure remains a considerable risk factor for the burden of disease in present-day China [1]. For instance, in 2013, only 3 out of 74 cities with ground-level air quality monitoring stations met China’s Ambient Air Quality Standard (GB 3095-2012). In contrast, this number increased to 222 out of 339 cities meeting the standard by 2024. Regarding water quality, the proportion of major river water sections categorized as “good” (Class I-III) and “poor” (inferior to Class V) was 71.7% and 9.0%, respectively, in 2013. By 2024, the proportion of “good” waters had risen to 92.4%, while that of “poor” waters had fallen to 0.3% [1]. Nevertheless, according to data from the Institute for Health Metrics and Evaluation (IHME), environmental and occupational risks were responsible for approximately 3.4 million deaths and 76.4 million disability-adjusted life years (DALYs) in China in 2021, accounting for approximately 28.7% of all deaths and 19.0% of all DALYs attributable to all risk factors [2].

Environmental monitoring across diverse outdoor and indoor settings is fundamental for accurate exposure assessment in environmental and public health research. Conventional approaches typically rely on fixed-site monitoring stations or samplers deployed at static locations [3,4,5,6,7,8]. These conventional methods often depend on high-end, stationary instruments or offline laboratory-based chemical analysis. For example, one study investigating the impact of different filters in home and office ventilation systems on adult cardiorespiratory function measured ambient PM_2.5_ and ozone levels at a nearby government monitoring station, while indoor levels were assessed using laboratory-grade instruments (e.g., TSI AM 510 SidePak for PM_2.5_ and 2B Tech Model 205 for ozone). Additionally, indoor volatile organic compounds (VOCs) and carbonyls were quantified through active sampling followed by mass spectrometric analysis [3]. However, the complexity and high cost of these conventional methods limit their mobility and scalability. Moreover, these approaches are often impractical for measuring accurate and continuous personal environmental exposures.

The rapid advancement of sensor technology in recent years has facilitated the development of numerous low-cost monitors and devices for environmental monitoring. Several well-known manufacturers, such as Plantower, Shinyei, Dylos, PurpleAir, MetOne, and Alphasense, produce such low-cost sensors and monitors [9]. The application of these devices has expanded globally to capture critical environmental events and complement regulatory networks. For instance, in Mexico, EMGA stations were deployed to record rapid increases in PM2.5 during fireworks celebrations [10]. In densely populated regions like Delhi, India, researchers have validated low-cost sensors for real-time PM_10_ monitoring, demonstrating their utility in resource-constrained environments [11]. Similarly, calibrated PurpleAir sensors were used to estimate wildfire smoke levels in California [12]. Beyond these ambient monitoring efforts, many recent studies have employed these portable monitors for personal exposure assessment. Chatzidiakou et al. used personal air quality monitors integrating multiple miniaturized sensors (e.g., for particulate matter, nitrogen oxides, and ozone) to improve the estimation of individual exposure doses [13]. Ma et al. used portable noise sensors to continuously measure the noise exposure levels of subjects [14]. Liu et al. used wearable ultraviolet radiation (UVR) sensors to assess the UVR exposure level of college students and pupils [15]. Compared to conventional devices, these mobile monitors are typically low-cost, compact, lightweight, and energy-efficient, enabling the collection of data with high spatiotemporal resolution. It is important to note that the definition of “low-cost” is relative and varies depending on the user and specific application. In this review, we adopt the definition outlined by the U.S. Environmental Protection Agency (EPA) and clarified by Morawska et al. [16], which refers to monitors or devices costing less than USD 2500.

Several previous reviews have examined the development and applications of portable low-cost sensors in environmental monitoring [16,17,18,19,20]. For example, Xu et al. reviewed the design of wireless sensor networks and their application in marine environmental monitoring [19]. Similarly, Morawska et al. focused on the validation, deployment, and data accessibility of low-cost sensors, alongside their applications [16]. Additionally, Salamone et al. systematically reviewed wearable devices for environmental monitoring in the built environment, with a focus on sensor development and limitations [17]. In summary, while previous reviews have provided valuable insights, they predominantly concentrate on the sensors themselves (e.g., design, validation) and often overlook the diversity of their real-world applications. Although a 2017 review explored the applications of miniaturized monitors for air pollution monitoring [20], it does not encompass the numerous relevant studies published since then. Moreover, its scope was limited to air pollutants, excluding other critical environmental factors such as water and soil pollution, microbes, noise, and light.

This review aims to provide a comprehensive synthesis of studies that employ portable low-cost sensors in real-world environmental monitoring campaigns. It encompasses a wide range of environmental factors (e.g., air, water, and soil pollution; microbes; noise; and light) across diverse settings (both outdoor and indoor). Therefore, this review aims to present a holistic picture of current research employing low-cost sensors for environmental monitoring and to offer guidance for the design of future studies and the selection of appropriate sensors.

## 2. Materials and Methods

### 2.1. Data Source and Search Strategy

Following a systematic approach adapted from a previous study [21], we conducted a literature search in the Web of Science for articles published between 1 January 1990 and 31 July 2025, focusing exclusively on studies conducted in China. The detailed search queries are provided in Table S1 (Supplementary Materials). In brief, the search strategy combined terms related to general “environmental factors” and specific factors of interest (i.e., air, water, and soil pollution; microbes; noise; and light) with keywords pertaining to “wearable”, “portable”, or “low-cost” sensors. All keywords and their relevant synonyms were searched within the topic field, which encompasses titles, abstracts, and author keywords. Ideally, the search strategy could be refined by incorporating terms like “field test” to better target real-world monitoring studies. However, no consistent terminology exists for “field testing” in the literature, as such studies may be conducted across diverse settings. Therefore, we initially retrieved a broader set of articles and manually screened them for eligibility based on the predefined criteria. Additionally, we performed a manual review of the reference lists within the included articles to identify and incorporate any additional studies that satisfied the eligibility criteria.

### 2.2. Eligibility Criteria

The eligibility criteria were defined as follows: (1) peer-reviewed original research articles with full text available; (2) articles published in the English language.; (3) studies conducting field tests or real-world monitoring campaigns; (4) studies carried out in China. Given the review’s focus on practical applications, studies primarily concerned with sensor development or laboratory-based performance evaluation were excluded. Furthermore, only studies using portable and low-cost sensors, as defined above, were selected. For studies employing Do-it-Yourself (DIY) sensor assemblies, the total cost of the core sensor components was required to be under USD 2500 to align with the low-cost definition.

The literature screening process involved multiple stages. First, one reviewer screened the titles and abstracts of all identified records against the eligibility criteria. Subsequently, a second reviewer independently assessed the full texts of the remaining articles. Finally, all reviewers collectively examined the final shortlisted literature to ensure consistency and quality. Any disagreements arising during the screening process were resolved through consensus discussion.

### 2.3. Study Inclusion

**Figure 1:**
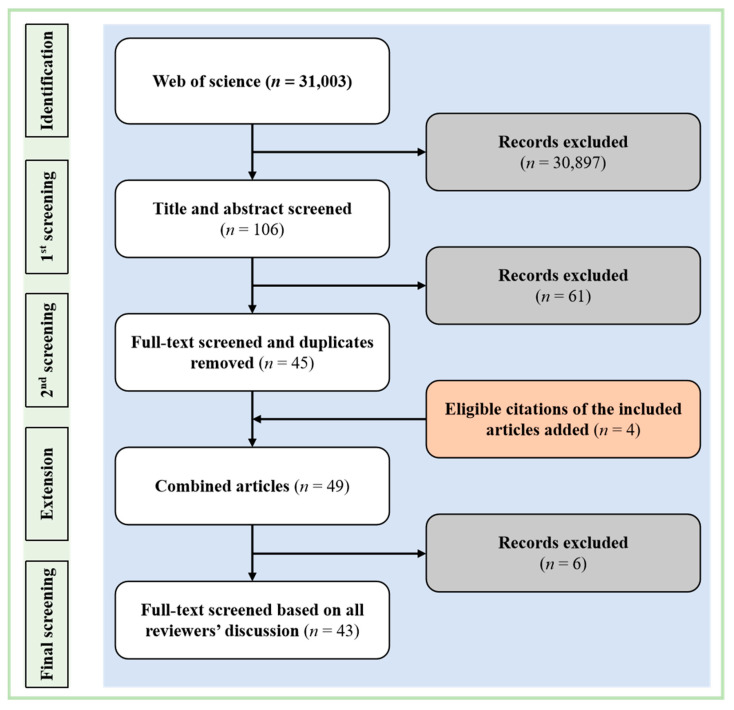
Flow chart of study inclusion: The diagram details the sequential phases of identification, screening, and eligibility assessment. Initial records were retrieved from the Web of Science database (*n* = 31,003). The screening process applied specific inclusion criteria: peer-reviewed original research conducted in China utilizing portable low-cost sensors for field monitoring. Key exclusion criteria were applied to remove studies focused solely on sensor development, laboratory-based performance evaluation without field ap-plication, or those not written in English, resulting in a final set of 43 eligible studies.

The study selection process is summarized in Figure 1. The initial database search yielded 31,003 records. Following the initial screening of titles and abstracts, 106 records were retained for full-text review. After a full-text assessment by a second independent reviewer, which led to the exclusion of non-qualifying and duplicate records, 45 articles remained. Additionally, a manual search of the reference lists from these 45 articles identified 4 further eligible publications, resulting in a total of 49 articles. A final collective review by all authors led to the exclusion of 6 additional articles. Consequently, a final total of 43 studies were included.

### 2.4. Analysis Strategy

We summarized the 43 included studies based on the environmental factor category of each study. Specifically, the studies were categorized into four groups, i.e., air pollution, noise, light and water pollution. For each subgroup, eligible studies were summarized in a table containing information on specific environmental factors, scenario, location, period of field tests, subjects, and sensor models. Furthermore, we discussed the challenges and opportunities in each subgroup. The review focused more on summarizing the methods of the reviewed studies, especially in the aspect of sensor application, rather than the results or conclusions. Consequently, the analysis aims to help guide future sensor-based studies on how to select low-cost environmental sensors and design field-based projects.

## 3. Results

### 3.1. Overview

**Figure 2:**
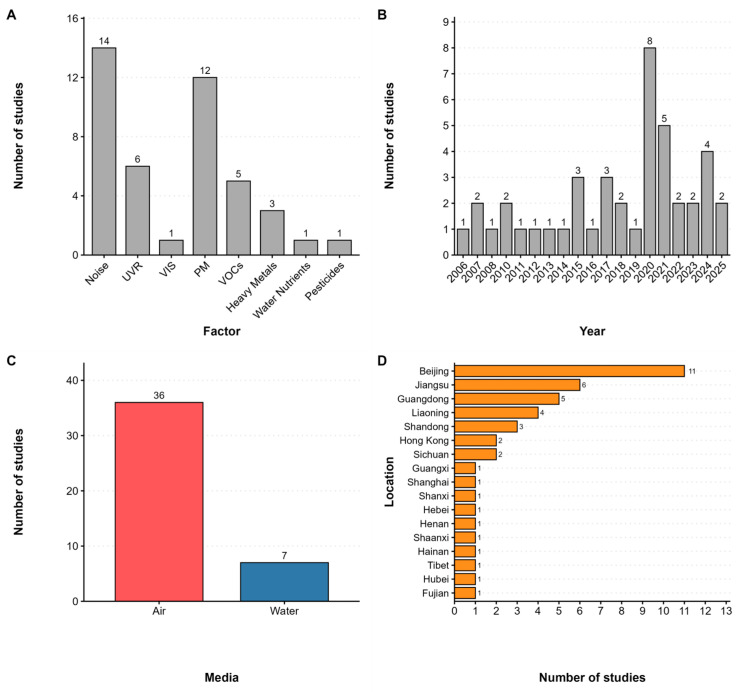
Overview of the literature characteristics: (**A**) The distribution of studies focused on different environmental factors; (**B**) The distribution of studies across different publication years; (**C**) The distribution of studies across different environmental media; (**D**) Geographical distribution of the studies.

**Figure 3:**
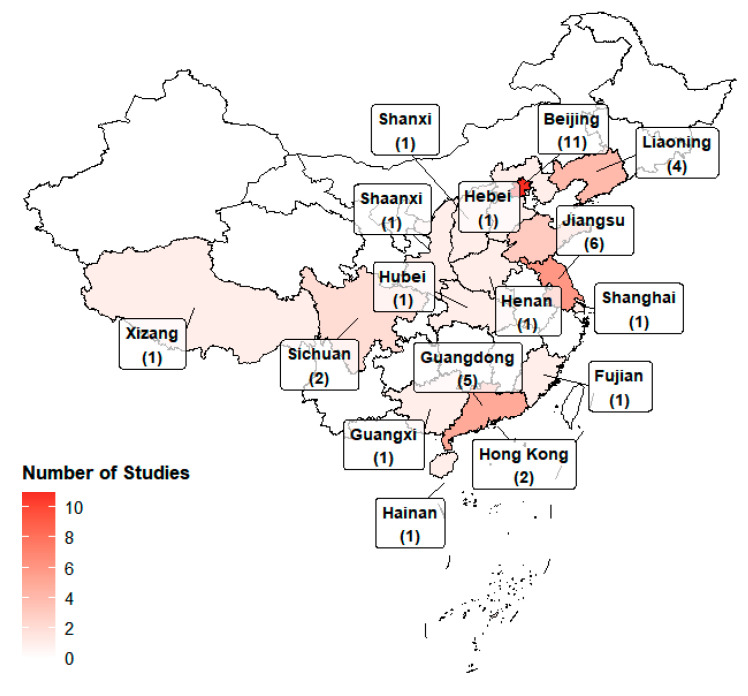
Geographic distribution of the 43 reviewed studies across China.

As summarized in Figure 2 and Table S3 (Supplementary Materials), the 43 studies include 17 on air pollution, 14 on noise, 7 on light, and 5 on water pollution. No eligible studies on soil pollution or microbes were found. Among the 17 studies on air pollution, twelve examined particulate matter (PM), and five examined total volatile organic compounds (TVOCs). No eligible studies were found for ozone, ammonia, radon, sulfur oxides, or a specific VOC. Regarding light, six studies measured UVR and one investigated visible light (VIS). No studies addressed electromagnetic or infrared radiation. In terms of environmental media, most studies (36 out of 43) focused on the atmospheric environment, with only seven addressing the aquatic environment. Thirty-three studies were published in the past ten years, while 22 were published in the past five years, indicating growing research interest in this field. Geographically, the studies were primarily conducted in Beijing (eleven papers) and Jiangsu (six papers), with the others in Liaoning, Shanghai, Guangdong, etc. The geographic distribution is visualized in Figure 3. As shown in the map, research efforts are heavily skewed towards economically developed regions in Eastern China (e.g., Beijing–Tianjin–Hebei, Yangtze River Delta and Guangdong province), while vast areas in Western and Central China remain largely under-investigated.

### 3.2. Air Pollution

In this section, we found 12 eligible studies on PM, and 5 on VOC, as summarized in Table 1 and Table 2.

One of the most significant threats in this generation is air pollution, impacting not only climate change but also public and individual health. Among them, PM, particles of variable but minimal diameter, penetrate the respiratory system via inhalation, causing respiratory and cardiovascular diseases, cancer, and so on [22,23,24]. Furthermore, nitrogen oxide, sulfur dioxide, VOCs, dioxins, and polycyclic aromatic hydrocarbons (PAHs) are all considered air pollutants harmful to humans [25]. As mentioned above, diseases from the above substances cause respiratory problems such as chronic obstructive pulmonary disease (COPD), asthma and lung cancer, and cardiovascular events [26].

Therefore, our primary goal is to find accurate and reliable detection tools. Traditional testing methods, while ensuring accuracy, often have the disadvantage of being expensive or inconvenient to carry. Low-cost, portable detection equipment is gradually maturing for application. The following paper will discuss the previous application of low-cost sensors from the perspective of several contaminants.

#### 3.2.1. Particulate Matter

PM_2.5_ (particulate matter with an aerodynamic diameter ≤ 2.5 μm) is a representative pollutant in China, with prolonged atmospheric residence times that significantly impact air quality and visibility. China’s annual average PM_2.5_ concentrations historically exceed those of many developed nations [27]. Epidemiological evidence firmly links PM_2.5_ exposure to increased risks of respiratory and cardiovascular morbidity and mortality [28,29,30,31,32,33]. PM_2.5_ monitoring is commonly categorized into indoor and outdoor environments, which exhibit distinct spatiotemporal patterns and health implications [26]. This section summarizes 12 studies on the application of portable low-cost sensors in China, which are shown in Table 1. To further illustrate the temporal characteristics of these studies, Figure S1 visualizes their timeline and duration stratified by monitoring scenarios.

Before detailing specific applications, it is essential to characterize the technology underpinning these studies. The majority of the reviewed campaigns employed commercial sensors (e.g., Plantower PMS series, Shinyei PPD, Nova SDS) that rely on the light scattering principle (specifically Mie scattering). While cost-effective, these sensors rely on assumed particle density profiles to convert optical signals into mass concentrations, which creates inherent challenges in selectivity. Quantitatively, the lower detection limit is typically around 1–5 µg/m^3^, with a dynamic range usually extending up to 500 or 1000 µg/m^3^, making them generally suitable for China’s ambient levels but less precise for clean environments [34,35]. A critical operational limitation is hygroscopic growth: at moderate humidity (50–80%), particles absorb water and scatter more light, potentially causing concentration to vary by as much as 20–50% if not algorithmically corrected [36]. Furthermore, regarding particle size discrimination, these optical sensors typically struggle to detect ultrafine particles (<0.1 μm)—which contribute little to mass but significantly to number concentration—thereby limiting their utility for assessing exposure to combustion-derived nanoparticles [37].

Despite these limitations, the flexibility of low-cost sensors enables novel approaches to outdoor air pollution monitoring. Traffic is a major source of outdoor PM_2.5_. Liu et al. demonstrated the use of low-cost sensors to monitor ambient PM and estimate traffic-related emission factors [38]. Similarly, another study by Liu et al. deployed a bicycle-based mobile monitoring system to assess near-road air quality [39]. Complementing fixed stations, which are costly to maintain and deploy densely, low-cost sensors can enhance spatial resolution. For instance, Chao et al. showed that integrating low-cost sensor networks with fixed stations significantly improves the spatiotemporal resolution of PM data, enabling more detailed pollution mapping and source identification [40]. Other studies have further exploited this spatial refinement at various scales. Wang et al. deployed sensors across a university campus, identifying distinct spatiotemporal variations and lower PM_2_._5_ levels compared to the urban background [41]. In a city-wide application, Liang et al. utilized a mobile network of sensors on taxis, coupled with big-data analytics, to map fine-scale pollution patterns and identify major emission sources [42]. Focusing directly on sources, Li et al. used a portable system to characterize ultrafine particle emissions from diesel trucks, quantifying significant reductions associated with improved emission standards and control technologies [43]. Gao et al. evaluated a low-cost PM sensor against reference instruments in a high-concentration urban setting in Xi’an [44]. Similarly, Bai et al. conducted a long-term field evaluation of a low-cost PM_2.5_ sensor in Nanjing [45]. Their findings, consistent with other studies, indicate that with proper calibration, low-cost sensors can achieve acceptable accuracy for ambient PM_2.5_ measurement.

In contrast to outdoor environments, indoor air pollution exhibits distinct spatiotemporal patterns and source profiles, which have been less quantified despite significant health implications. Shen et al. employed low-cost sensors in a Beijing apartment to quantify indoor PM_2.5_ levels and identify major sources [46]. Their findings identified outdoor infiltration and cooking as the primary PM_2.5_ sources, with cooking being a more dominant contributor. Indoor PM_2.5_ concentrations correlated with, but were generally lower than, ambient levels. In rural China, household air pollution from solid fuel combustion poses a severe health risk. Men et al. conducted a four-month monitoring campaign using low-cost sensors to track indoor PM_2.5_ from coal burning in rural households. They analyzed temporal dynamics, indoor–outdoor relationships, and quantified the contribution of indoor sources [47]. Together, these studies demonstrate that low-cost sensors are a valuable tool for characterizing indoor exposure with high spatiotemporal resolution, crucial for identifying key sources and exposure patterns.

Beyond ambient concentration, personal exposure is more directly relevant to health. Chatzidiakou et al. deployed multi-pollutant personal sensors to 251 participants in Beijing to measure individual exposure [13]. Similarly, Yang and Zhao used a wearable monitoring system to demonstrate substantial discrepancies between personal PM_2_._5_ exposure and ambient station measurements among college students [48]. These studies underscore the potential of low-cost sensors for quantifying truly personal exposure, which often differs from ambient concentrations.

**Table 1:** Studies focusing on PM.

Study	Location (China)	Scenario	Period	Subject	Exposure Measurement	Number of Records
[38]	Beijing	Traffic	2018	9 locations	PMS1003, BAM-1020, EC9830	1 week for each subject
[39]	Changzhou, Jiangsu	Traffic	2015	n. a	Shinyei PPD42NS TGS 2201	Few days
[40]	Xinxiang, Henan	Outdoor	2017	144 locations	XHAQSN-808 model	1 year for each subject
[41]	Beijing	Outdoor	2023	5 locations	CGDN1	35 days for each subject
[42]	Rizhao, Shandong	Traffic	2019–2020	102 taxis	SDS019–25	12 months for each taxi
[43]	Guangzhou, Guangdong	Traffic	2023	10 trucks	ELPI (Dekati, Finland)	33 km for each truck
[44]	Xi’an, Shaanxi	Outdoor	2013	8 locations	Shinyei PPD42NS	7 days for each location
[45]	Nanjing, Jiangsu	Outdoor	2015–2017	1 location	Shinyei PPD42NS BAM-1020	2 years for the location
[46]	Beijing	Indoor	2020	15 indoor sites	PM-Model-II	10 days for each subject
[47]	Hebei	Indoor	2021	70 rural homes	Model 5030 Synchronized Hybrid Ambient Realtime Particulate Monitor	4 months for each subject
[13]	Beijing	Outdoor	2016	251 participants	PAM	1 month for each participant
[48]	Beijing	Indoor	2023	96 participants	PMS7003	2 days for each participant

#### 3.2.2. VOCs

VOCs are a broad group of carbon-based chemicals that evaporate easily at room temperature [49]. In the context of low-cost sensing, these are often reported as specific compounds (e.g., benzene, toluene, ethylbenzene, and xylene, collectively known as BTEX) or as TVOC, which represents the aggregate concentration typically measured in parts per billion (ppb) or micrograms per cubic meter (µg/m^3^). Typical TVOC baseline levels in non-industrial indoor environments generally range from 50 to several hundred ppb, whereas outdoor background levels are usually lower, though traffic emissions can cause significant local spikes [50]. Many VOCs are recognized as human carcinogens [51]. They are common indoor air pollutants with documented short- and long-term adverse health effects. Short-term exposure can cause irritation to the eyes, nose, and throat, while chronic exposure is linked to systemic toxic effects [52]. These significant health risks underscore the urgent need for accurate, portable, and intelligent monitoring methods. Two primary low-cost sensing principles are widely used: Metal Oxide Semiconductor (MOS) and Photoionization Detectors (PID). MOS sensors are dominant in low-cost applications due to their affordability, yet they suffer from significant selectivity limitations; they react non-specifically to various reducing gases (e.g., alcohols, CO) and are prone to baseline drift caused by humidity and temperature [53]. Their detection limit typically ranges from 0.1 to 1 ppm, which is often insufficient for monitoring trace-level ambient VOCs in non-industrial settings. In contrast, PID sensors offer superior sensitivity (down to 1–10 ppb) and faster response time [54]. However, they remain limited by the ionization potential of the UV lamp (typically 10.6 eV), meaning they cannot distinguish between specific compounds in a complex mixture without upstream separation steps. Navigating these technological characteristics, this section reviews 5 studies that utilized portable low-cost sensors for VOC monitoring in China, which are shown in Table 2.

**Table 2:** Studies focusing on VOCs.

Study	Location (China)	Scenario	Period	Subject	Exposure Measurement	Number of Records
[55]	Guangdong	Ambient air monitoring	2019	VOCs in air	Photoionization detector (PID)	Multiple ambient air samples
[56]	Guangdong	Food safety	2022	Vegetable samples	Screen-printed electrode (SPE) aerometric sensor	53 records (one per vegetable sample)
[57]	Hubei	Vehicle emission monitoring	2023	Gasoline and diesel vehicles	Portable emission measurement system (PEMS)	Multiple vehicles
[58]	Beijing	Artwork preservation	2017	Formaldehyde in artworks	Colorimetric sensor (Spectrophon, Rehovot, Israel)	Multiple monitoring tests
[59]	Chengdu, Sichuan	Multiple detection	2011	Samples	Dual-channel optical sensor	20 types of VOCs

Traditional VOC analysis often requires offline sample collection and laboratory processing, which is time-consuming. Low-cost portable sensors offer a solution for real-time, on-site detection. Pang et al. integrated a commercial low-power photoionization detector (PID) into a portable, low-energy gas chromatography (GC) system for ambient hydrocarbon-like VOC analysis [55]. It was used for detecting hydrocarbon-like VOCs in ambient air. Zhang et al. employed a low-cost handheld detector to successfully identify formaldehyde contamination in 53 vegetable samples [56]. Similarly, Niu et al. used a portable system with low-cost VOC sensors for real-time vehicle exhaust monitoring, effectively capturing compositional variations under different driving conditions [57]. These field-based applications demonstrate the capability of low-cost VOC sensors for accurate real-time monitoring in diverse settings.

The connectivity of low-cost sensors (e.g., via Bluetooth or Wi-Fi) enables the creation of large-scale networks and intelligent data platforms. Formaldehyde, a common and concerning VOC, has garnered increasing attention. Real-time data collection and intelligent platforms for formaldehyde detection could significantly benefit industries such as interior decoration and construction. Some researchers have developed smartphone-connected, low-cost sensors for formaldehyde detection. For instance, Zilberstein et al. developed an intelligent platform using a low-cost formaldehyde sensor, which transmitted real-time data to a smartphone via Bluetooth [58]. This system was deployed to monitor formaldehyde levels near artworks in Beijing’s Summer Palace. The sensor data showed strong agreement with commercial devices, validating their reliability. This approach presents a promising strategy for future large-scale data collection and sharing.

Beyond single-analyte devices, multi-analyte sensors capable of simultaneous detection have also been developed. Such multi-analyte sensors can significantly improve monitoring efficiency. Hu et al. developed a novel dual-channel sensor based on surface photovoltage (SPV) and photoluminescence (PL), which could identify 20 different volatile compounds under UV induction [59]. The sensor also successfully distinguished between complex mixtures like wine, liquor, and vinegar. This work points to a promising direction for future research in multi-analyte sensing.

### 3.3. Noise Pollution

Noise, defined as “unwanted sound”, is a significant environmental pollutant with substantial adverse effects on human health. The hazards can be broadly classified into two categories: auditory and non-auditory effects. Auditory effects primarily involve noise-induced hearing loss (NIHL) [60]. According to the 2010 Global Burden of Disease [61], 13 billion people are affected by hearing loss, and investigators rated hearing loss as the 13th most significant contributor to the global number of years lived with disability (YLD) (19.9 million years, 2.6% of the total). The World Health Organization (WHO) estimates that 10% of the global population is exposed to sound pressure levels capable of causing NIHL. Hearing loss in about half of these individuals is attributable to exposure to intense noise [62]. Non-auditory effects encompass a wider range of health issues, including annoyance, sleep disturbance, impaired cognitive performance, and cardiovascular, endocrine, and psychiatric disorders [60,63]. According to WHO [64], DALYs due to environmental noise in EU member states and other Western European countries are 903,000 for sleep disturbance, 587,000 for annoyance, 61,000 for ischemic heart disease, 45,000 for cognitive impairment in children, and 22,000 for tinnitus. This substantial public health burden underscores the necessity for effective monitoring and exposure assessment. To meet this demand, portable sound level meters and personal dosimeters have been widely adopted.

Technologically, the devices reviewed in this section (e.g., SLM-25, AWA5610) typically rely on electret condenser microphones. Unlike studio-grade equipment, these portable sensors are designed for field portability, generally operating within a dynamic range of 30–130 dB. While professional models (e.g., Aihua) offer higher precision, the lower-cost consumer-grade units often employed in citizen science face limitations such as a relatively high noise floor (>30 dBA) and potential sensitivity drift over time. With these operational characteristics in mind, this section reviews 14 studies employing such devices for noise monitoring in China, which are shown in Table 3. The studies involved occupational (4 studies), daily life (5), industrial plant (2), traffic (1), restaurant (1), and built environment (1) scenarios. Based on deployment mode, the studies are categorized as either “carry-on” (personal) or “fixed location”. Carry-on devices enable more accurate assessment of personal noise exposure. For instance, a series of studies used portable sensors (e.g., SLM-25) co-located with GPS smartphones to collect real-time, individual-level noise exposure and spatiotemporal trajectory data, analyzing noise levels across different activities [14,65,66,67,68]. Fixed location deployments, conversely, are used to characterize site-specific noise levels. For example, Wang et al. monitored noise levels across 23 operating rooms in a tertiary hospital [69]. Other monitored sites included textile factories, restaurants, and areas surrounding high-speed trains [70,71,72,73,74]. Among the studies, the subjects ranged from 43–659, and the monitoring periods ranged from hours to two days.

### 3.4. Light Pollution

#### 3.4.1. Ultraviolet Radiation

Human exposure to UVR is a well-established cause of adverse health effects, including skin cancer, cataracts, pterygium, and immunosuppression [15,78]. Skin cancers are among the most common malignancies globally, with over one million new cases diagnosed annually [79]. In the United States alone, they account for nearly 15,000 deaths and over $3 billion in annual medical costs [80]. UVR is estimated to be a causative factor in approximately 65% of melanoma cases and 90% of non-melanoma skin cancers [80]. While skin cancer risk varies by skin phenotype, UVR-induced eye diseases, such as cataracts, represent a universal health concern. Cataracts are a leading cause of blindness worldwide, responsible for over 16 million cases. Ocular exposure to UVR is a significant risk factor for developing both cataracts and pterygium [78].

From a sensing standpoint, the UV monitors deployed in these studies (e.g., PD204 series) generally employ photodiodes paired with optical filters to isolate specific UVA or UVB bands. Unlike broad-band radiometers, these low-cost sensors often face challenges in spectral mismatch, where the sensor’s sensitivity curve does not perfectly align with the erythemal action spectrum (human skin sensitivity) [81]. Furthermore, accurate measurement requires proper cosine correction to account for light arriving from different angles, a feature sometimes compromised in miniaturized wearable designs.

With these technological characteristics in context, this subsection reviews applications of UVR sensors in China, which are shown in Table 4. The six eligible studies were divided into two categories: five measured personal UVR exposure doses, and one monitored ambient UVR levels. One study assessed the seasonal variation in UVR exposure doses among 62 students in Shenyang [15]. The remaining four studies, from the same research group, employed manikins to quantify ocular UVR exposure doses [78,82,83,84]. The single study in the second category examined diurnal and seasonal variations in ambient UVR at the northern edge of the Tibetan Plateau [85]. Although portable sensors were used in all studies, the deployment strategy varied. Most studies involved fixed-point monitoring (e.g., sensors mounted on manikin eyes) rather than wearable deployment on participants. Only one study employed a wearable approach, positioning sensors on participants’ upper arms [15]. The studies utilized sensors with different spectral sensitivities: SUB-T type sensors measured broad-spectrum UVA and UVB, while PD204A and PD204B sensors were specific to UVA and UVB, respectively. Monitoring durations across these studies ranged from several days to years.

#### 3.4.2. Visible Light

Inappropriate illumination levels—both excessive and insufficient—have been documented to negatively affect human physiology and comfort [86,87]. Only one eligible study investigating visible light was identified, the details of which are included in Table 4.

Tu et al. used portable sensors to investigate human responses to different illumination levels and CO_2_ concentrations within an underground shelter environment [86]. Their study involved 24 male participants, each undergoing two 3.5-h exposure sessions in a basement setting. Illumination levels were continuously monitored, while questionnaires were administered to assess subjective thermal responses and acute health symptoms.

### 3.5. Water Pollution

Water is an essential resource, yet many regions in China face significant threats from water pollution [88]. Pollutants such as heavy metals and pesticides pose direct and indirect risks to human health and ecosystem integrity. Low-cost sensors enable the development of portable platforms for real-time, multi-analyte water quality monitoring. This section reviews 5 studies on the application of portable low-cost sensors for water quality monitoring in China, which are shown in Table 5.

Regarding detection principles, the reviewed studies utilize diverse approaches tailored to specific analytes, primarily falling into two categories: optical methods and electrochemical sensing. While optical methods offer high sensitivity, they are susceptible to turbidity interference in complex water matrices [89]. Electrochemical sensors provide rapid responses but often struggle with electrode fouling during continuous immersion [90]. Building upon these principles, researchers have developed innovative platforms for automation. For instance, building upon colorimetric analysis, researchers have developed low-cost platforms for the automated, on-site detection of phosphate and nitrite. While phosphate is an essential agricultural nutrient, elevated concentrations (e.g., >0.2 mg/L) can cause eutrophication, severely impacting aquatic ecosystems [91]. Nitrite is highly toxic to aquatic biota and is a potential human carcinogen. To address these needs, Lin et al. developed a low-cost sensor for the automated, on-site monitoring of phosphate and nitrite in agricultural waters [92]. The device utilizes multiple reagents for sequential colorimetric assays, enabling high-throughput, automated screening of multiple water samples in the field. Low-cost optical sensors are also being advanced for the rapid field monitoring of heavy metals. For instance, Chang et al. developed a portable system integrating a novel CMC-MOF membrane with a handheld fluorescence spectrometer, achieving sensitive on-site detection of trace Cr (VI) in groundwater (detection limit: 3.72 ppb) [93].

Visual detection methods offer the distinct advantage of user-friendliness, enabling application by non-specialists. This approach facilitates cost-effective, principle-based optical detection of diverse contaminants, such as the herbicide trifluralin. Excessive trifluralin exposure is associated with adverse health effects including hepatorenal toxicity, allergic reactions, and immunotoxicity [94]. To address this, Farshchi et al. developed a wearable electrochemical glove sensor using conductive silver nano-inks for in-situ monitoring of trifluralin residues on various surfaces [95]. Similarly, Chen et al. designed a glove-based sensor incorporating fluorescent carbon dots from cyanobacteria for the visual detection of Pb^2+^ in water via fluorescence quenching [96]. Visual detection strategies have also been applied to mercury. Li et al. developed a low-cost test strip based on filter paper for the visual, immediate, and quantitative detection of Hg^2+^ in water [97]. This sensor demonstrated high analytical performance for detecting Hg^2+^ in diverse matrices, including water, seafood, and human urine. Collectively, these studies highlight the potential of low-cost sensors as powerful tools for rapid, real-time, and portable detection of water pollution.

**Table 5:** Studies focusing on water pollution.

Study	Location (China)	Scenario	Period	Subject	Exposure Measurement	Number of Records
[92]	Xiamen, Fujian	Agriculture water monitoring	2018	Agricultural water samples	Low-cost automatic colorimetric sensor	Multiple field samples
[93]	Guangxi	Water pollution	2024	Groundwater samples from contaminated sites	Portable laser-induced fluorescence (LIF) system with CMC–MOF membrane probe	3 types of groundwater samples
[95]	Nanjing, Jiangsu	Environmental pollution monitoring	2021	Trifluralin residues on various substrates	Wearable glove electrochemical sensor	Multiple samples
[96]	Jiangsu	Water pollution	2023	Different water samples	Wearable glove sensor	3 types of water samples
[97]	Shandong	Environmental pollution monitoring	2020	Different samples	Low-cost fluorescent probe (2TS)	Multiple samples from each matrix

## 4. Discussion

This review has identified several overarching limitations in the current body of research, indicating that the application of portable low-cost sensors in large-scale environmental monitoring still faces substantial hurdles. This section critically discusses these challenges, focusing on the scope of environmental factors monitored, sensor shortcomings, and the study scale. Subsequently, we propose promising future research directions to address these gaps.

### 4.1. Scope of Environmental Factors Monitored

The reviewed studies demonstrate a pronounced imbalance in the environmental factors monitored. Research is heavily concentrated on air and noise pollution, with significantly less attention paid to water and light pollution, and a near-total absence of studies on soil and biological contaminants.

Within the domain of air pollution, it is notable that no eligible studies were found for ozone. It is important to clarify that this reflects the specific scope of our review rather than a total absence of technology. For instance, low-cost ozone sondes are routinely deployed in China (e.g., for satellite data verification in Kunming [98]) for meteorological vertical profiling. However, these applications focus on upper-atmosphere physics and were excluded from our analysis, which strictly prioritizes portable monitoring for human exposure assessment.

What’s more, the scarcity of field studies on soil and biological contaminants involves specific technological bottlenecks regarding stability and matrix interference, rather than a total lack of sensing options. For soil monitoring, Ion-Selective Electrodes (ISEs) have been proposed as low-cost solutions. However, quantitative reviews [99,100] highlight that unlike air sensors, ISEs in abrasive soil slurries suffer from rapid membrane fouling and leaching. Technically, this results in significant potential drift (often exceeding 1–2 mV/h) and limits the operational lifespan of unmodified sensors to often less than a week without recalibration. Furthermore, selectivity coefficients present a major hurdle; for example, high concentrations of chloride ions in soil can inherently interfere with nitrate detection, causing measurement errors that require complex compensation algorithms unsuitable for simple low-cost nodes. Similarly, for biological agents, while aptamer-based biosensors demonstrate femtomolar-level sensitivity in buffered laboratory solutions [101,102], they lack the robustness for field use. The primary barrier is environmental instability: bioreceptors are prone to irreversible denaturation at ambient temperatures (e.g., >30 °C), and effective detection typically requires labor-intensive sample pretreatment (filtration and pH adjustment) to prevent non-specific binding from complex environmental matrices. This contradicts the “portable” requirement of sensors reviewed here. Consequently, analysis of these contaminants still largely relies on traditional laboratory-based methods, as true in-situ detections such as directly inserting a sensor into soil or water for immediate, quantitative readouts of specific pollutants is not yet widely feasible.

### 4.2. Sensor Shortcomings

The reviewed literature points to several critical areas requiring improvement in current sensor technology: (1) The performance and accuracy of these sensors have not been thoroughly evaluated. Although most studies have calibrated the sensors with specialized equipment before using them, it is still doubtful whether they can operate reliably for long periods or maintain accuracy under certain environments (e.g., high temperature, high humidity). Only a few studies comparing the sensors with professional equipment have been reported in the literature. For example, Cui et al. [85] compared sensor measurements with satellite data. Therefore, it is not yet possible to blindly trust the data measured by these sensors. (2) Some of the sensors are large and not portable enough. For example, Lin et al. explored the automated detection of phosphate and phosphite in agricultural water environments. Although using low-cost sensors to build on-site automatic detection machines dramatically reduces costs and time compared to traditional laboratory-based detection methods such as liquid chromatography, the size of the devices is still not portable enough (280 mm in length), and there is room for further optimization. A genuinely portable sensor should be as watch-like as the sensors applied by Liu and Zilberstein et al. [15,58]. (3) Some of the sensors are disposable and cannot be reused. For example, a paper sensor was designed by Zhang et al. to directly visualize and measure mercury content in water [103]. Although it has the advantages of being low-cost, lightweight, high sensitivity, and arbitrarily tailored, it can only be used once and still causes many inconveniences. (4) The data transmission method is not convenient enough. Currently, most of the sensor data is stored on the device itself, and some early studies even required the subject to record the sensor readings in person [15]. This results in the need for periodic retrieval of the sensors, which results in inconvenient experimental procedures and geographical limitations. Therefore, the ideal data transfer method would be for the sensor to upload the collected data regularly or in real time to smartphone software, which would be uploaded to the cloud through the smartphone, where relevant personnel could download and analyze it. The bracelet-type formaldehyde sensor developed by Zilberstein et al. enables the transfer of the collected data to a smartphone through a Bluetooth channel [58].

### 4.3. Study Scale and Spatial Coverage

A recurring limitation across the reviewed literature is the predominance of short-term, small-scale studies. As shown in the results, monitoring durations are typically brief (e.g., [44] lasted only one week), and sample sizes are generally small (e.g., only 24 participants in [86]). Such limited cohorts undermine the statistical power and the ability to capture long-term environmental trends, which is particularly critical for investigating health outcomes.

Overcoming these scale limitations is epidemiologically vital because it directly addresses the “exposure measurement error” inherent in centralized monitoring. While regulatory stations (e.g., the US EPA instruments) provide gold-standard data for regional compliance, they inherently fail to capture personal exposure heterogeneity [104]. Crucially, individuals are mobile and typically spend over 80% of their time in indoor micro-environments (e.g., homes, offices) [105], which centralized outdoor stations cannot monitor. Consequently, relying solely on sparse, outdoor central stations fails to reflect the actual dynamic dosage received by individuals. The primary justification for low-cost sensors is their ability—when properly calibrated to maintain performance within acceptable limits—to travel with the subject or be deployed in these specific micro-environments. They provide the spatiotemporal granularity needed to link specific peaks (e.g., cooking fumes, traffic intersections) to health outcomes. Furthermore, long-term sensor deployment is strictly necessary to assess cumulative exposure for chronic disease epidemiology, moving beyond static ambient averages to capture the complex, longitudinal exposure profiles of individuals.

Technologically, low-cost sensors offer a distinct advantage in deployment of geometry and network density. While regulatory stations typically operate at a macro-scale with sparse distribution (e.g., one station per tens of square kilometers) to represent regional background levels, low-cost sensors allow for hyper-local monitoring. The reviewed studies demonstrate diverse geometries that significantly enhance resolution. For instance, Chao et al. [40] utilized a stationary grid geometry with 144 sensors to achieve high-density coverage, whereas Liang et al. [42] adopted a mobile sensing approach using 102 taxis to dynamically map pollution at the street level. These high-density configurations shift the representativeness from “district-wide” averages to the identification of specific “hotspots” in traffic canyons or industrial fence-lines that centralized networks overlook.

However, this gain in spatial resolution entails a critical cost–benefit trade-off regarding data quality. Regulatory stations provide high stability and accuracy but at high capital costs, limiting their spatial granularity. Conversely, low-cost sensors facilitate broad spatial coverage at a fraction of the cost but are prone to lower accuracy, drift, and cross-sensitivity. Therefore, achieving reliable large-scale monitoring requires accepting higher data uncertainty or implementing robust calibration strategies (e.g., using sparse regulatory stations to calibrate dense sensor networks). The current scarcity of such large-scale studies highlights that balancing the trade-off between coverage quantity and data quality remains a substantial logistic and technical hurdle.

### 4.4. Future Research Directions

To translate potential into widespread practical impact, future research should move beyond proof-of-concept demonstrations. While air pollution monitoring is the most mature application, studies often remain siloed, focusing on data collection in limited areas without developing generalizable models or integration frameworks. A critical next step is the creation of integrated, scalable sensing systems. This entails advancing robust, wireless data transmission protocols and building centralized data platforms. Such infrastructure is essential for achieving real-time, large-scale and personal environmental monitoring, moving from isolated studies to a cohesive network that can inform public health and policy decisions.

## 5. Conclusions

This review examined the application of portable low-cost sensors for monitoring environmental pollution in China. By synthesizing existing studies—detailing the types of sensors used, their purposes, and deployment methodologies, this work aims to present researchers with the current status of the field, while also discussing the strengths and limitations of both the sensors and the studies themselves. The evidence shows that these sensors significantly enhance the spatiotemporal resolution of human exposure data and serve as a vital complement to regulatory equipment, offering great versatility across various experimental designs. However, challenges remain in areas such as accuracy, miniaturization, and data transmission. It is anticipated that ongoing technological advancements will address these issues, making the large-scale application of portable low-cost sensors a reality. Importantly, while this review focuses on China, the findings carry global relevance. The unique conditions of rapid urbanization, diverse pollution sources, and high population density in China provide a critical testbed for these technologies. The universal challenges identified here, including sensor stability in complex environments, the necessity for local calibration, and deployment trade-offs, mean that the lessons learned offer valuable insights for designing robust monitoring strategies worldwide, particularly in other rapidly developing regions with comparable resource constraints and pollution profiles.

## Figures and Tables

**Table 3:** Studies focusing on noise pollution.

Study	Location (China)	Scenario	Period	Subject	Exposure Measurement	Number of Records
[14]	Beijing	Daily life	2017–2018	117 adults living in the study area	SLM-25 Sound Level Meters (Gain Express Holdings Ltd., HK, China)	48 h for each subject
[65]	Beijing	Daily life	2017–2018	117 adults living in the study area	SLM-25 Sound Level Meters (Gain Express Holdings Ltd., HK, China)	48 h for each subject
[66]	Beijing	Daily life	2017–2018	117 adults living in the study area	SLM-25 Sound Level Meters (Gain Express Holdings Ltd., HK, China)	48 h for each subject
[67]	Guangzhou, Guangdong	Daily life	2018–2019	156 adults living in the study area	SLM-25 Sound Level Meters (Gain Express Holdings Ltd., HK, China)	48 h for each subject
[68]	Guangzhou, Guangdong	Daily life	2018–2019	92 adults living in the study	SLM-25 Sound Level Meters (Gain Express Holdings Ltd., HK, China)	48 h for each subject
[69]	Beijing	Occupational	2015–2016	23 operating rooms	Personal noise dosimeters (Aihua, Model AWA5610B, Hangzhou, China)	Not mentioned
[70]	Nanjing, Jiangsu	Occupational	2006	659 female workers	HS6288 sound level meter	One time for each subject
[71]	Jinan, Shandong	Industrial plants	Not mentioned	Centrifugal pump	AWA14423L type microphone (Hangzhou Aihua Instruments Co., Ltd., Hangzhou, China)	One time for each subject
[72]	Chengdu, Sichuan	Traffic	Not mentioned	High-speed train	Microphone array (B&K WA-0890-F)	One time for each subject
[73]	Hong Kong	Built environment	Not mentioned	203 measurement sites	Sound level meter (B&K 2236)	8 h for each subject
[74]	Hong Kong	Restaurant	Not mentioned	12 restaurants	Sound level meter (B&K 2236)	Two or three times for each subject
[75]	Shanghai	Occupational	Not mentioned	43 surgeons	Sound level meter (Control Company, Friendswood, TX)	One time for each subject
[76]	Taiyuan, Shanxi	Occupational	2005	124 overhead-traveling crane drivers	Personal noise dosimeters (AIHUA Instruments Model AWA5610e, Hangzhou, China)	8 h for each subject
[77]	Beijing	Industrial plants	2013	270 workers	ASV5910 personal sound exposure meter	Half an hour for each subject

**Table 4:** Studies focusing on light pollution.

Study	Location (China)	Scenario	Period	Subject	Exposure Measurement	Number of Records
[15]	Shenyang, Liaoning	Daily life	2001–2002	32 pupils and 30 undergraduate medical students	UV sensor (Model: SUB-T, Toray, Techno Inst)	224 h for each subject
[78]	Sanya, Hainan	Outdoor	2009	Manikins	UV sensor (SUB-T; Toray Industries, Tokyo, Japan)	5 days for each subject
[82]	Shenyang, Liaoning	Outdoor	2004–2007	Manikins	UV sensor (SUB-T; Toray Industries, Tokyo, Japan)	Not mentioned
[83]	Shenyang, Liaoning	Outdoor	Not mentioned	n. a	UV sensor (SUB-T; Toray Industries, Tokyo, Japan)	n. a
[84]	Shenyang, Liaoning	Outdoor	2005–2007	Manikins	UV sensor (SUB-T; Toray Industries, Tokyo, Japan)	18 days for each subject
[85]	Tibet	Plateau environment	2001–2003	n. a	PD204A and PD204B sensors (Macam Photometrics Ltd., Scotland)	n. a
[86]	Nanjing, Jiangsu	Underground refuge chamber	2019	24 males	Metrel MI6201	7 h for each subject

## Data Availability

All data needed to evaluate the conclusions in the paper are present in the paper and/or the Supplementary Materials. Additional data related to this paper is available on request.

## Supplementary Materials

The following supporting information can be downloaded at: https://www.mdpi.com/article/10.3390/s26010085/s1, Figure S1: Timeline and duration of field monitoring campaigns in the included PM studies; Table S1: Literature search strategy; Table S2: Technical specifications of representative low-cost sensors identified in the reviewed studies; Table S3: Summary of the included studies.

## Abbreviations

The following abbreviations are used in this manuscript:

DALYs	Disability-Adjusted Life Years
YLDs	Years Lived with Disability
GC	Gas Chromatography
PM	Particulate Matter
PM_2.5_	Particulate Matter (with a kinetic diameter of less than or equal to 2.5 µm)
TVOCs	Total Volatile Organic Compounds
PAHs	Polycyclic Aromatic Hydrocarbons
VIS	Visible Light
UVR	Ultraviolet Radiation
