# Supplementary material for: Portable Low-Cost Sensors for Environmental Monitoring in China: A Comprehensive Review of Application, Challenges, and Opportunities
Source: Sensors (Basel). 2025 Dec 22;26(1):85. doi: 10.3390/s26010085 (PMC12787898; doi:10.3390/s26010085)
Supplement: Supplementary file 1 [file sensors-26-00085-s001.zip › sensors-3970271-supplementary.pdf]

sensors

MDPI

Review

Portable Low-Cost Sensors for Environmental Monitoring in China: A Comprehensive Review of Application, Challenges, and Opportunities

Chunhui Yang, Ruiyuan Wu, Yang Zhao * and Jianbang Xiang *

School of Public Health (Shenzhen), Sun Yat-sen University, Shenzhen 518107, China; yangchh28@mail2.sysu.edu.cn (C.Y.); wury28@mail2.sysu.edu.cn (R.W.)
* Correspondence: xiangjb@mail.sysu.edu.cn (J.X.); zhaoy393@mail.sysu.edu.cn (Y.Z.):

The supplementary materials contain one figure and three tables.

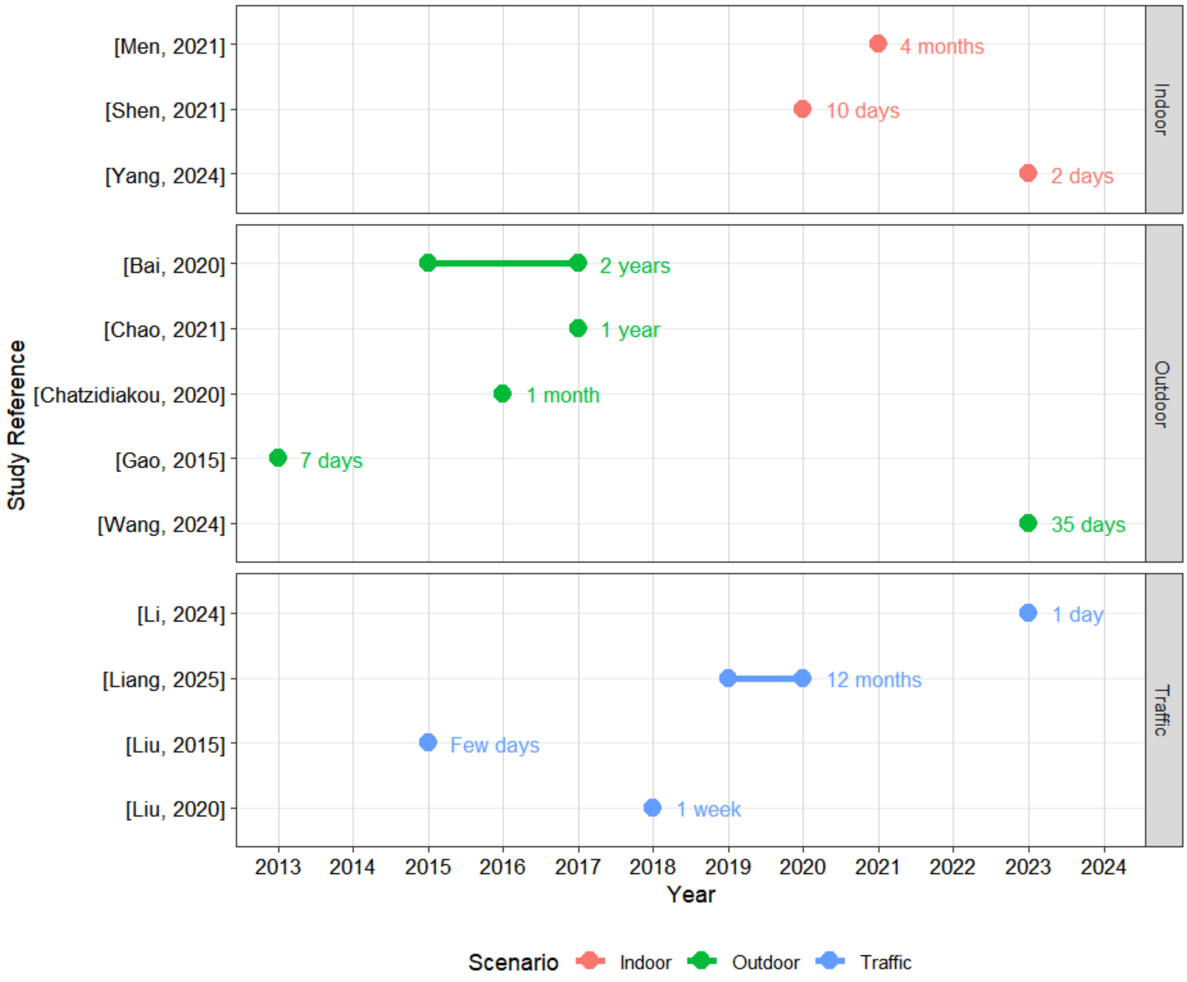

Figure S1. Timeline and duration of field monitoring campaigns in the included PM studies: the studies are stratified by monitoring scenarios (Indoor, Outdoor, and Traffic). Horizontal segments represent multi-year monitoring periods, while points indicate studies conducted within a single year. Text annotations specify the reported duration of each campaign. References included in this

 https://doi.org/10.3390/s26010085

timeline: Men, 2021 [23]; Shen, 2021 [22]; Yang, 2024 [31]; Bai, 2020 [29]; Chao, 2021 [25]; Chatzidiakou, 2020 [27]; Gao, 2015 [28]; Wang, 2024 [32]; Li, 2024 [33]; Liang, 2025 [30]; Liu, 2015 [26]; Liu, 2020 [24].

Table S1. Literature search strategy.

Database	Query	Records
Web of Science	TS = (environmental factor* OR nois* OR temperature OR radiat* OR UV OR infrared OR visible light OR VIS OR illumination OR air pollut* OR ozone OR organic* OR VOC* OR PM OR partic* OR biolog* OR water pollu* OR soil pollut*) AND TS = (wearable OR portable OR low-cost) AND TS = (sensor* OR monitor*) AND (TS = China OR AD = China)	31003

Table S2. Technical specifications of representative low-cost sensors identified in the reviewed studies.

Sensor	Target Pollutant	Principle	Detection Range	Resolution
PMS1003	PM	Light scattering	0 – 500 μg/m³	1 μg/m³
Shinyei PPD42NS	PM	Light scattering	0 – 800 μg/m³	1 μg/m³
CGDN1	PM	Light scattering	0 – 500 μg/m³	1 μg/m³
SDS019 – 25	PM	Light scattering	0 – 2000 μg/m³	1 μg/m³
PMS7003	PM	Light scattering	0 – 500 μg/m³	1 μg/m³
SLM-25 Sound Level Meters	Noise	Electret condenser microphones	30 – 130 dB	0.1 dB
AWA5610B	Noise	Electret condenser microphones	50 – 140 dB	n.a
AWA5610e	Noise	Electret condenser microphones	50 – 140 dB	n.a
HS6288 sound level meter	Noise	Electret condenser microphones	30 – 135 dB	0.1 dB
AWA14423L	Noise	Electret condenser microphones	18 – 140 dB	n.a
ASV5910 personal sound exposure meter	Noise	Electret condenser microphones	40 – 140 dB	n.a
PD204	UVR	Photodiodes	n.a	n.a
Metrel MI6201	UVR	Photodiodes	n.a	n.a

Table S3. Summary of the included studies.

Study	Title	Year	Location	Media	Environmental factor
[1]	Evaluation of noise hazard during the holmium laser enucleation of prostate	2017	Shanghai	Air	Noise
[2]	A cross-sectional study in a tertiary care hospital in China: noise or silence in the operating room	2017	Beijing	Air	Noise
[3]	Associations of blood pressure and arterial compliance with occupational noise exposure in female workers of textile mill	2007	Nanjing, Jiangsu	Air	Noise
[4]	Experimental Study on Radiation Noise Frequency Characteristics of a Centrifugal Pump with Various Rotational Speeds	2018	Jinan, Shandong	Air	Noise
[5]	Assessment of personal noise exposure of overhead-traveling crane drivers in steel-rolling mills	2007	Taiyuan, Shanxi	Air	Noise
[6]	Health impacts of construction noise on workers: A quantitative assessment model based on exposure measurement	2016	Beijing	Air	Noise
[7]	An Experimental Investigation into the Difference in the External Noise Behavior of a High-Speed Train between Viaduct and Embankment Sections	2022	Chengdu, Sichuan	Air	Noise
[8]	Noise in restaurants: Levels and mathematical model	2014	Hong Kong	Air	Noise
[9]	Are the noise levels acceptable in a built environment like Hong Kong?	2015	Hong Kong	Air	Noise
[10]	Geographic Ecological Momentary Assessment (GEMA) of environmental noise annoyance: the influence of activity context and the daily acoustic environment	2020	Guangzhou,Guangdong	Air	Noise
[11]	Assessing personal noise exposure and its relationship with mental health in Beijing based on individuals' space-time behavior	2020	Beijing	Air	Noise
[12]	Associations of co-exposures to air pollution and noise with psychological stress in space and time: A case study in Beijing, China	2021	Beijing	Air	Noise
[13]	Examining the effects of mobility-based air and noise pollution on activity satisfaction	2020	Beijing	Air	Noise
[14]	Association between real-time noise exposure in broader activity contexts and job satisfaction: Evidence from Guangzhou, China	2025	Guangzhou,Guangdong	Air	Noise

https://doi.org/10.3390/xxxxx

Study	Title	Year	Location	Media	Environmental factor
[15]	Individual solar-UV doses of pupils and undergraduates in China	2006	Shenyang, Liaoning	Air	UVR
[16]	Diurnal and seasonal variations of UV radiation on the northern edge of the Qinghai-Tibetan Plateau	2008	Xizang	Air	UVR
[17]	Diurnal Variations in Solar Ultraviolet Radiation at Typical Anatomical Sites	2010	Shenyang, Liaoning	Air	UVR
[18]	Diurnal Variations in Solar Ultraviolet Radiation on Horizontal and Vertical Plane	2010	Shenyang, Liaoning	Air	UVR
[19]	Skin ultraviolet exposure dosimetry using rotating manikin	2012	Shenyang, Liaoning	Air	UVR
[20]	Solar UV Exposure at Eye Is Different from Environmental UV: Diurnal Monitoring at Different Rotation Angles Using a Manikin	2013	Sanya, Hainan	Air	UVR
[21]	Study on human responses under different CO2 concentration and illuminance in underground refuge chamber	2020	Nanjing, Jiangsu	Air	VIS
[22]	Temporal and spatial variation of PM2.5 in indoor air monitored by low-cost sensors	2021	Beijing	Air	PM
[23]	Contributions of internal emissions to peaks and incremental indoor PM(2.5)in rural coal use households	2021	Hebei	Air	PM
[24]	An experimental application of laser-scattering sensor to estimate the traffic-induced PM(2.5)in Beijing	2020	Beijing	Air	PM
[25]	Integrating Fixed Monitoring Systems with Low-Cost Sensors to Create High-Resolution Air Quality Maps for the Northern China Plain Region	2021	Xinxiang, Henan	Air	PM
[26]	A Bicycle-borne Sensor for Monitoring Air Pollution near Roadways	2015	Chang-zhou,Jiangsu	Air	PM
[27]	Using low-cost sensor technologies and advanced computational methods to improve dose estimations in health panel studies: results of the AIRLESS project	2020	Beijing	Air	PM
[28]	A distributed network of low-cost continuous reading sensors to measure spatiotemporal variations of PM2.5 in Xi'an, China	2015	Xi'an,Shaanxi	Air	PM
[29]	Long-term Field Evaluation of Low-cost Particulate Matter Sensors in Nanjing	2020	Nanjing, Jiangsu	Air	PM

https://doi.org/10.3390/xxxxx

Study	Title	Year	Location	Media	Environmental factor
[30]	Understanding the origins of urban particulate matter pollution based on high-density vehicle-based sensor monitoring and big data analysis	2025	Rizhao, Shandong	Air	PM
[31]	A real-time personal PM2.5 exposure monitoring system and its application for college students	2024	Beijing	Air	PM
[32]	Characteristics of PM2.5 and CO2 Concentrations in Typical Functional Areas of a University Campus in Beijing Based on Low-Cost Sensor Monitoring	2024	Beijing	Air	PM
[33]	Exploring ultrafine particle emission characteristics from in-use light-duty diesel trucks in China using a portable measurement system	2024	Guangzhou,Guangdong	Air	PM
[34]	Differences in compositions and effects of VOCs from vehicle emission detected using various methods	2023	Hubei	Air	VOCs
[35]	Low-cost photoionization sensors as detectors in GC x GC systems designed for ambient VOC measurements	2019	Gungdong	Air	VOCs
[36]	A miniaturized sensor for detection of formaldehyde fumes	2017	Beijing	Air	VOCs
[37]	Practical and Efficient: A Pocket-Sized Device Enabling Detection of Formaldehyde Adulteration in Vegetables	2022	Gungdong	Water	VOCs
[38]	UV-Induced Surface Photovoltage and Photoluminescence on n-Si/TiO2/TiO2:Eu for Dual-Channel Sensing of Volatile Organic Compounds	2011	Chengdu, Sichuan	Water	VOCs
[39]	Bithiophene-based fluorescent sensor for highly sensitive and ultrarapid detection of Hg2+ in water, seafood, urine and live cells	2020	Shandong	Water	Hg
[40]	Low-Cost Automatic Sensor for in Situ Colorimetric Detection of Phosphate and Nitrite in Agricultural Water	2018	Xiamen,Fujian	Water	Phosphate/Nitrite
[41]	Trifluralin recognition using touch-based fingertip: Application of wearable glove-based sensor toward environmental pollution and human health control	2021	Nanjing, Jiangsu	Water	Trifralin
[42]	Wearable Design for Occupational Safety of Pb2+ Water Pollution Monitoring Based on Fluorescent CDs	2023	Jiangsu	Water	Pb
[43]	Metal-organic framework membrane-based probe for on-site and sensitive detection of Cr(VI) in groundwater using a portable system	2024	Guangxi	Water	Cr

References

1. Xu, H.; Chen, Y.B.; Gu, M.; Chen, Q.; Wang, Z. Evaluation of noise hazard during the holmium laser enucleation of prostate. *Bmc Urol.* **2017**, *17*.
2. Wang, X.X.; Zeng, L.; Li, G.; Xu, M.; Wei, B.; Li, Y.; Li, N.; Tao, L.Y.; Zhang, H.; Guo, X.Y., et al. A cross-sectional study in a tertiary care hospital in china: Noise or silence in the operating room. *Bmj Open* **2017**, *7*.
3. Ni, C.H.; Chen, Z.Y.; Zhou, Y.; Zhou, J.W.; Pan, J.J.; Liu, N.; Wang, J.; Liang, C.K.; Zhang, Z.Z.; Zhang, Y.J. Associations of blood pressure and arterial compliance with occupational noise exposure in female workers of textile mill. *Chin. Med. J.* **2007**, *120*, 1309–1313.
4. Guo, C.; Gao, M.; Lu, D.Y.; Guan, H.J. Experimental study on radiation noise frequency characteristics of a centrifugal pump with various rotational speeds. *Appl. Sci. -Basel* **2018**, *8*.
5. Zeng, L.; Chai, D.L.; Li, H.J.; Lei, Z.; Zhao, Y.M. Assessment of personal noise exposure of overhead-traveling crane drivers in steel-rolling mills. *Chin. Med. J.* **2007**, *120*, 684–689.
6. Li, X.D.; Song, Z.Y.; Wang, T.; Zheng, Y.; Ning, X. Health impacts of construction noise on workers: A quantitative assessment model based on exposure measurement. *J. Clean. Prod.* **2016**, *135*, 721–731.
7. Li, M.X.; Deng, T.S.; Wang, D.; Xu, F.; Xiao, X.B.; Sheng, X.Z. An experimental investigation into the difference in the external noise behavior of a high-speed train between viaduct and embankment sections. *Shock Vib.* **2022**, *2022*.
8. To, W.M.; Chung, A.W.L. Noise in restaurants: Levels and mathematical model. *Noise Health* **2014**, *16*, 368–373.
9. To, W.M.; Mak, C.M.; Chung, W.L. Are the noise levels acceptable in a built environment like hong kong? *Noise Health* **2015**, *17*, 429–439.
10. Zhang, X.; Zhou, S.H.; Kwan, M.P.; Su, L.L.; Lu, J.W. Geographic ecological momentary assessment (gema) of environmental noise annoyance: The influence of activity context and the daily acoustic environment. *Int. J. Health Geogr.* **2020**, *19*.
11. Ma, J.; Li, C.J.; Kwan, M.P.; Kou, L.R.; Chai, Y.W. Assessing personal noise exposure and its relationship with mental health in beijing based on individuals' space-time behavior. *Environ. Int.* **2020**, *139*.
12. Tao, Y.H.; Kou, L.R.; Chai, Y.W.; Kwan, M.P. Associations of co-exposures to air pollution and noise with psychological stress in space and time: A case study in beijing, china. *Environ. Res.* **2021**, *196*.
13. Ma, J.; Rao, J.W.; Kwan, M.P.; Chai, Y.W. Examining the effects of mobility-based air and noise pollution on activity satisfaction. *Transp. Res. Part D-Transp. Environ.* **2020**, *89*.
14. Song, J.Y.; Zhou, S.H.; Kwan, M.P.; Liao, Y.T.; Liu, D.; Zhang, X. Association between real-time noise exposure in broader activity contexts and job satisfaction: Evidence from guangzhou, china. *CITIES* **2025**, *161*.
15. Liu, Y.; Ono, M.; Yu, D.; Wang, Y.; Yu, J. Individual solar-uv doses of pupils and undergraduates in china. *J. Expo. Sci. Environ. Epidemiol.* **2006**, *16*, 531–537.
16. Cui, X.; Gu, S.; Zhao, X.; Wu, J.; Kato, T.; Tang, Y. Diurnal and seasonal variations of uv radiation on the northern edge of the qinghai-tibetan plateau. *Agric. For. Meteorol.* **2008**, *148*, 144–151.
17. Hu, L.-W.; Gao, Q.; Xu, W.-Y.; Wang, Y.; Gong, H.-Z.; Dong, G.-Q.; Li, J.-H.; Liu, Y. Diurnal variations in solar ultraviolet radiation at typical anatomical sites. *Biomed. Environ. Sci.* **2010**, *23*, 234–243.
18. Hu, L.W.; Gong, H.Z.; Yu, D.J.; Gao, Q.; Gao, N.; Wang, M.; Yan, Y.; Wang, Y.; Yu, J.; Liu, Y. Diurnal variations in solar ultraviolet radiation on horizontal and vertical plane. *Iran. J. Public Health* **2010**, *39*, 70–81.
19. Hu, L.; Gao, Q.; Xu, W. Skin ultraviolet exposure dosimetry using rotating manikin. *China Public Health* **2012**, *28*, 1207–1209.
20. Hu, L.; Gao, Q.; Gao, N.; Liu, G.; Wang, Y.; Gong, H.; Liu, Y. Solar uv exposure at eye is different from environmental uv: Diurnal monitoring at different rotation angles using a manikin. *J. Occup. Environ. Hyg.* **2013**, *10*, 17–25.
21. Tu, Z.; Geng, S.; Li, Y.; Iop. In Study on human responses under different co2 concentration and illuminance in underground refuge chamber. In Proceedings of the 4th International Workshop on Renewable Energy and Development (IWRED), Electr Network, 2020 Apr 24-26, 2020; Electr Network, 2020.
22. Wu, S.; Li, Z.; Zhang, J.; Wu, X.; Deng, X.; Liu, Y.; Zhou, J.; Zhi, C.; Yu, X.; Choy, W.C.H., et al. Low-bandgap organic bulk-heterojunction enabled efficient and flexible perovskite solar cells. *Adv. Mater.* **2021**, *33*.
23. Men, Y.T.; Li, J.P.; Liu, X.L.; Li, Y.J.; Jiang, K.; Luo, Z.H.; Xiong, R.; Cheng, H.F.; Tao, S.; Shen, G.F. Contributions of internal emissions to peaks and incremental indoor pm(2.5)in rural coal use households. *Environ. Pollut.* **2021**, *288*.
24. Liu, X.T.; Zhao, Q.; Zhu, S.C.; Peng, W.J.; Yu, L. An experimental application of laser-scattering sensor to estimate the traffic-induced pm(2.5)in beijing. *Environ. Monit. Assess.* **2020**, *192*.

25. Chao, c.Y.; Zhang, h.; Hammer, m.; Zhan, y.; Kenney, d.; Martin, r.V.; Biswas, p. Integrating fixed monitoring systems with low-cost sensors to create high-resolution air quality maps for the northern china plain region. *Acs Earth Space Chem.* **2021**, *5*, 3022–3035.
26. Liu, x.F.; Li, b.; Jiang, a.M.; Qi, s.X.; Xiang, c.S.; Xu, n.; IEEE. In *A bicycle-borne sensor for monitoring air pollution near roadways*, IEEE International Conference on Consumer Electronics - Taiwan (ICCE-TW 2015), Taipei, TAIWAN, Jun 06-08, 2015; Taipei, TAIWAN, 2015; pp 166-167.
27. Chatzidiakou, L.; Krause, A.; Han, Y.; Chen, W.; Yan, L.; Popoola, O.A.M.; Kellaway, M.; Wu, Y.; Liu, J.; Hu, M., et al. Using low-cost sensor technologies and advanced computational methods to improve dose estimations in health panel studies: Results of the airless project. *J. Expo. Sci. Environ. Epidemiol.* **2020**, *30*, 981–989.
28. Gao, M.L.; Cao, J.J.; Seto, E. A distributed network of low-cost continuous reading sensors to measure spatiotemporal variations of pm2.5 in xi'an, china. *Environ. Pollut.* **2015**, *199*, 56–65.
29. Bai, L.; Huang, L.; Wang, Z.L.; Ying, Q.; Zheng, J.; Shi, X.W.; Hu, J.L. Long-term field evaluation of low-cost particulate matter sensors in nanjing. *Aerosol Air Qual. Res.* **2020**, *20*, 242–253.
30. Liang, Y.H.; Wang, X.H.; Dong, Z.Z.; Wang, X.F.; Wang, S.D.; Si, S.C.; Wang, J.; Liu, H.Y.; Zhang, Q.Z.; Wang, Q. Understanding the origins of urban particulate matter pollution based on high-density vehicle-based sensor monitoring and big data analysis. *URBAN Clim.* **2025**, *59*.
31. Yang, W.N.; Zhao, B. A real-time personal pm2.5 exposure monitoring system and its application for college students. *Build Simul-China* **2024**, *17*, 1531–1539.
32. Wang, Q.Q.; Ao, R.X.; Chen, H.W.; Li, J.L.; Wei, L.F.; Wang, Z.F. Characteristics of pm2.5 and co2 concentrations in typical functional areas of a university campus in beijing based on low-cost sensor monitoring. *ATMOSPHERE* **2024**, *15*.
33. Li, D.; Wu, D.Y.; Gui, X.L.; Liao, S.D.; Zhu, M.N.; Yu, F.; Zheng, J.Y. Exploring ultrafine particle emission characteristics from in-use light-duty diesel trucks in china using a portable measurement system. *Env. Res* **2024**, *263*.
34. Niu, Z.Z.; Kong, S.F.; Zheng, H.; Hu, Y.; Zheng, S.R.; Cheng, Y.; Yao, L.Q.; Liu, W.; Ding, F.; Liu, X.Y., et al. Differences in compositions and effects of vocs from vehicle emission detected using various methods. *Environ. Pollut.* **2023**, *333*.
35. Pang, x.B.; Nan, h.J.; Zhong, j.P.; Ye, d.Q.; Shaw, m.D.; Lewis, a.C. Low-cost photoionization sensors as detectors in gc x gc systems designed for ambient voc measurements. *Sci. Total Environ.* **2019**, *664*, 771–779.
36. Zilberstein, G.; Zilberstein, R.; Zilberstein, S.; Maor, U.; Baskin, E.; Zhang, S.; Righetti, P.G. A miniaturized sensor for detection of formaldehyde fumes. *Electrophoresis* **2017**, *38*, 2168–2174.
37. Zhang, H.; Wu, Z.; Zhi, Z.; Gao, W.; Sun, W.; Hua, Z.; Wu, Y. Practical and efficient: A pocket-sized device enabling detection of formaldehyde adulteration in vegetables. *Acs Omega* **2022**, *7*, 160–167.
38. Hu, J.; Jiang, X.M.; Wu, L.; Xu, K.L.; Hou, X.D.; Lv, Y. Uv-induced surface photovoltage and photoluminescence on n-si/tio2/tio2:Eu for dual-channel sensing of volatile organic compounds. *Anal. Chem.* **2011**, *83*, 6552–6558.
39. Li, C.P.; Niu, Q.F.; Wang, J.G.; Wei, T.; Li, T.D.; Chen, J.B.; Qin, X.Y.; Yang, Q.X. Bithiophene-based fluorescent sensor for highly sensitive and ultrarapid detection of hg2+ in water, seafood, urine and live cells. *Spectrochim. Acta Part A-Mol. Biomol. Spectrosc.* **2020**, *233*.
40. Lin, B.C.; Xu, J.; Lin, K.N.; Li, M.P.; Lu, M. Low-cost automatic sensor for in situ colorimetric detection of phosphate and nitrite in agricultural water. *Acs Sens.* **2018**, *3*, 2541–2549.
41. Farshchi, F.; Saadati, A.; Kholafazad-Kordasht, H.; Seidi, F.; Hasanzadeh, M. Trifluralin recognition using touch-based fingertip: Application of wearable glove-based sensor toward environmental pollution and human health control. *J. Mol. Recognit.* **2021**, *34*.
42. Chen, H.; Xu, H.; Zhang, Y.D.; Gu, S.G.; Wang, D.D. Wearable design for occupational safety of pb2+ water pollution monitoring based on fluorescent cds. *AUTEX Res. J.* **2023**, *23*, 403–408.
43. Chang, X.Y.; Gao, N.S.; Meng, G.P.; Zhen, L.P.; Guo, W.T.; Zhang, P.; Dai, S.J.; Wang, B.D. Metal-organic framework membrane-based probe for on-site and sensitive detection of cr(vi) in groundwater using a portable system. *Chem. Eng. J.* **2024**, *493*.
